# Method for conducting micro-abrasion wear testing of materials in oscillating sliding

**DOI:** 10.1016/j.mex.2022.101703

**Published:** 2022-04-16

**Authors:** Cesar David Resendiz-Calderon, Irving Cázares-Ramírez, Diana Laura Samperio-Galicia, Leonardo Israel Farfan-Cabrera

**Affiliations:** Tecnologico de Monterrey, Escuela de Ingeniería y Ciencias, Ave. Eugenio Garza Sada 2501, N.L., Monterrey 64849, Mexico

## Abstract

Micro-abrasion is a kind of wear occurring on materials´ surfaces by action of three-body hard particles existing at the sliding interface. It is consistently reproduced by a popular laboratory method well-known as ball cratering test. Characteristically, it consists of wearing off a material sample by loading it against a ball that rotates continuously and unidirectionally under controlled conditions. A slurry containing a certain amount of hard micro-sized particles is dropped uninterruptedly onto the ball to get the particles enters the sliding contact by the ball´s rotary effect. The present method proposes a variant to the classic micro-abrasion methodology. It comprises changing the ball motion from a unidirectional to an oscillating for testing by considering new test parameters such as sliding arc lengths and frequency, which provides more realistic sliding conditions and wear rates for materials to be used in mechanical applications involving reciprocating and oscillating sliding.

## Highlights

• A method for conducting micro-abrasion testing of materials in oscillating sliding is provided.
• Oscillating parameters for micro-abrasion testing are given.
• The method includes parameters for reproducing rolling abrasion and grooving in oscillating sliding.

## Graphical abstract

**Figure:**
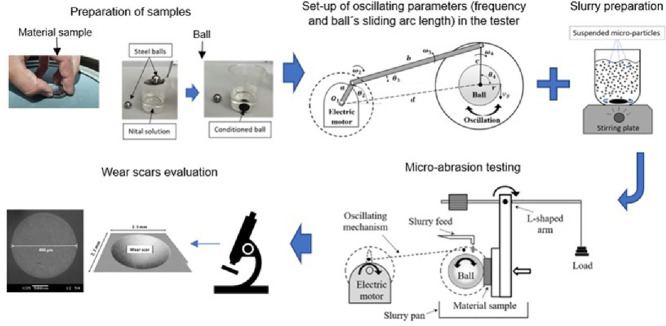

Specifications table

**Table:** 

Subject area:	Mechanical engineering; Materials characterization
More specific subject area:	Tribology
Method name:	Micro-abrasion wear of materials in oscillating sliding
Name and reference of original method:	A novel tester to examine micro-abrasion of materials in oscillating sliding contact – The case study of a total knee replacement biomaterial. - Resendiz-Calderon, C. D., Farfan-Cabrera, L. I., & Cázares-Ramírez, I. (2021). A novel tester to examine micro-abrasion of materials in oscillating sliding contact – The case study of a total knee replacement biomaterial. Wear, 203661. 10.1016/j.wear.2021.203661
Resource availability:	N.A.

## Background

**Fig. 1:**
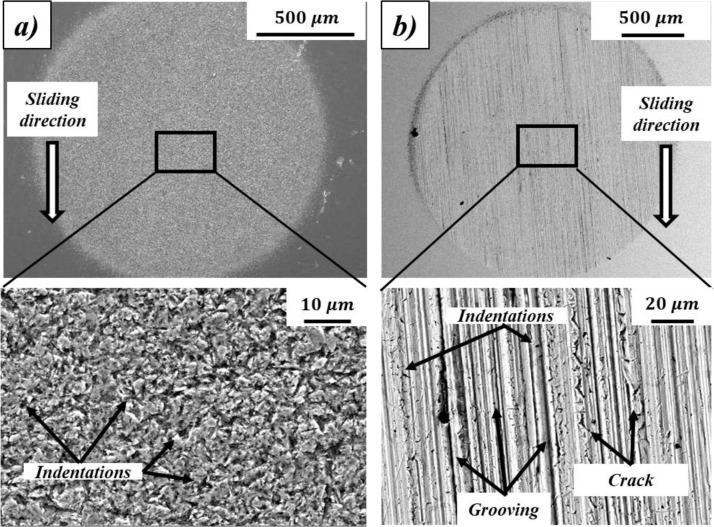
Examples of SEM images from micro-abrasion wear scars with different wear mechanism: (a) rolling abrasion; (b) grooving.

The micro-abrasion or ball-cratering test has become very popular for the characterization of wear resistance of a wide range of materials, namely, bulk materials (metals, polymers and ceramics) [1], [2], [3], [4], [5], [6], hard coatings (PVD and CVD) [1,[7], [8], [9], [10]], elastomers (silicone, neoprene, viton and ethylene-propylene-diene-monomer) [11], etc., under the specific mechanisms of rolling abrasion and grooving, as illustrated in Fig. 1a,b, which are caused by the action of hard micro-particles at the interface under controlled conditions. Both mechanisms can be reproduced by this test mainly varying load and the abrasive type and concentration [12]. Rolling abrasion is generated by high amounts of hard micro-particles at the sliding interface, which tend to roll during the relative motion of the surfaces producing micro-indentations and micro-cracking as the main wear patterns in the scar. It is typically associated with low loads and/or high abrasive slurry concentrations. Otherwise, grooving is an abrasion type caused by few hard micro-particles existing at the sliding interface, which are fixed at the ball surface and slide against the countersurface generating mostly micro-scratches [12]. It is commonly reproduced with high loads and/or low abrasive slurry concentrations. The uniqueness of this test is that the features of micro-abrasion mechanisms are consistently reproduced to form small and measurable wear craters with well-defined geometries, as those illustrated in Fig. 1a,b, in a short time, which cannot be achieved in other tribotesters or by other methods.

Fundamentally, the original micro-abrasion test is to generate wear with specific patterns of micro-abrasion on small (e.g., 100 to 400 mm^2^ surface areas, and 1 to 5 mm thickness) flat or curved material samples. Wear of the sample is produced by continuous and unidirectional sliding of a rotating ball that is loaded against a material sample at a predefined load while a slurry containing a certain amount of hard micro-sized particles is dropped uninterruptedly onto the ball to get into the sliding contact by the ball´s rotary effect. Load applied, speed, ball cycles and slurry feed rate can be varied in the test [6]. Balls can be made of a wide range of materials as desired for the testing. However, AISI 52100 steel (equivalent materials: ASTM A1031 Grade E52100, SAE 52100, DIN 100Cr6, ISO 102Cr8, AFNOR 100Cr6, etc.) is the most common for conventional micro-abrasion characterization of materials. The ball should be modified superficially to have a certain roughness (pitting) for promoting suitable engagement of the particles to generate damage to the sample, and thus, achieving consistent micro-abrasion wear rate results [13,14]. In other words, reproducible results are obtained with pre‐conditioned balls with slight surface pitting, and the use of such balls is recommended for routine testing [13,14]. This process should not have significant effect on the ball hardness. The roughness of these ‘modified’ steel balls is suggested to be 350 ± 70 nm Ra [13]. In order to preserve an appropriate ball´s pitted surface during the test, the ball must be rotated transversally each certain number of cycles. It is because sliding produces wear on the ball surface reducing the pitting produced purposely at the beginning of the test [14].

All the above testing characteristics are maintained in the proposed method in this work, but new parameters (i.e., oscillation frequency, arc lengths and ball transversal rotation) of consideration for the novel variant of the test (oscillating motion of the ball instead of one unidirectional) are included. The method, various results with the suggested parameters and evidence of repeatability are detailed in a previous reported research work [15] to demonstrate the robustness of the method. Hence, the method provided in this document includes the details of the tester capabilities required and the main parameters to be considered for achieving testing of micro-abrasion in oscillating siding contacts. In addition, a case study, including some laboratory results by applying the proposed method and parameters, is included.

## Method details

### Mechanical setup

**Fig. 2:**
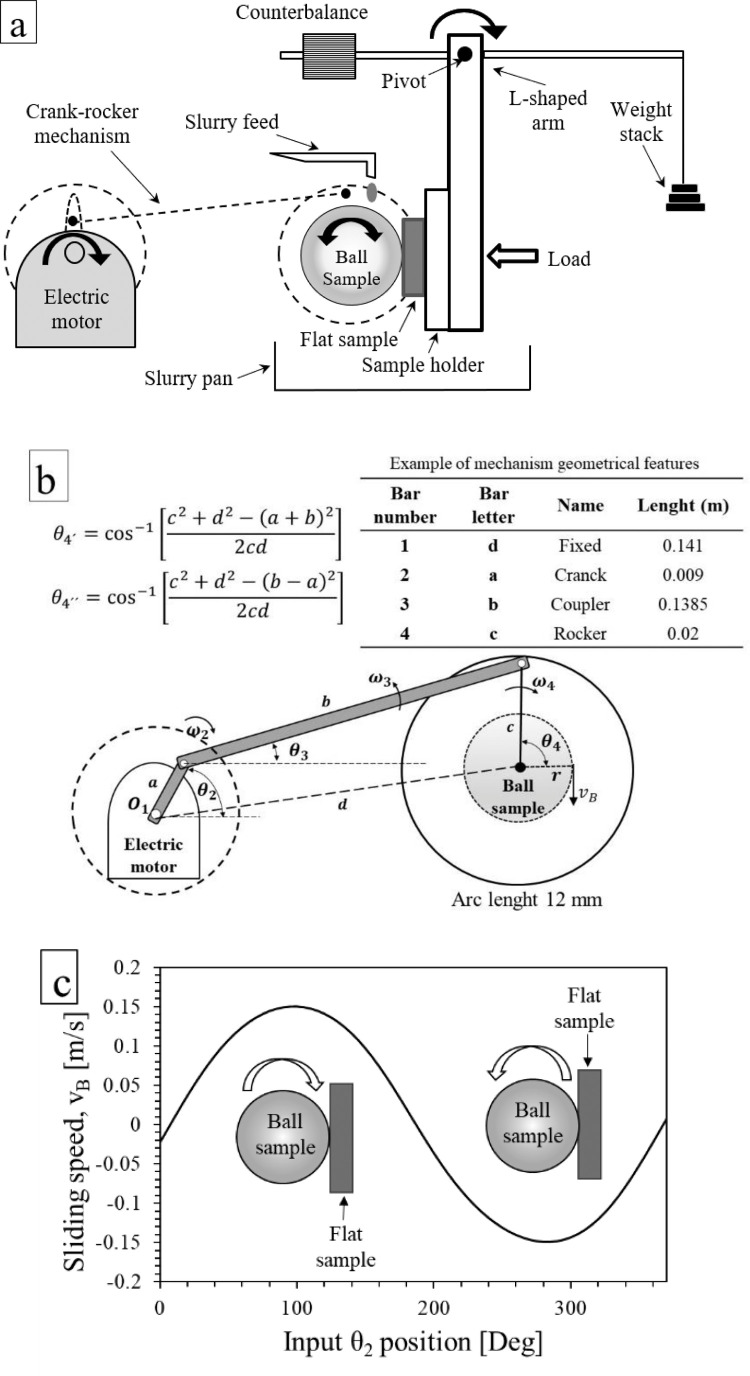
(a) Schematic diagram of a micro-abrasion tester configurated to reproduce oscillating ball motion; (b) example of geometrical features for a four-bars mechanism; (c) ball´s sliding speed, $v_{B}$, function resulted by using the geometrical features.

To conduct the method, a micro-abrasion tester capable of providing an oscillating motion of the ball, as the example proposed in the schematic illustration in Fig. 2a., is required. The tester must be capable to hold a 25.4 mm diameter steel ball sample, preferably, since it is the traditional ball diameter used for micro-abrasion tests [6,12]. However, balls with different diameter can be also used for the testing. The ball should be clamped between two coaxial driving shafts, which must be rotated by an electric motor through a mechanism able to generate oscillating motion to the ball. The motor must be controlled at a constant speed (between 0.5 ± 0.1 and 31.5 ± 0.5 rad/s) and certain ball cycles accurately. The speed should be controlled at a predefined value because variations could alter the wear process kinematics during the test. The ball cycles should be counted and controlled to generate accurately a predefined sliding distance. The material specimen, which can be flat or curved, should be hold in a plate (sample holder) located at the bottom of the pivoted L-shaped arm. A counterbalance is needed to provide equilibrium to the arm before applying the load selected. Preferably, the load should be applied by using dead-weights positioned at the end of the horizontal lever of the arm and measured by a force sensor, or by another system allowing the application of a defined constant load during the test. The slurry should be fed by using a peristaltic pump with controlled flux while a container should be used to collect the residual slurry. To provide the tester with an oscillating motion mode for the ball, a crank-rocker (four-bars) mechanism can be used, as that diagram illustrated in Fig. 2a,b. The mechanism involves 3 physical bars (labeled as “a”, “b” and “c”) and a fourth bar (labeled as “d”), which refers to the distance between the fixed points of bars “a” and “c”. This simple mechanical arrangement allows the transformation of rotation provided by the electric motor into oscillating motion of the ball sample to operate at predefined frequency and sliding arc length by controlling the mechanism parameters and the electric motor speed. The steel ball´s angular speed, $\omega_{4}$, in oscillating motion can be determined by means of a vector loop equation using the parameters for a four-bar mechanism configuration:

(1) $$ja\omega_{2}e^{j\theta_{2}}+jb\omega_{3}e^{j\theta_{3}}-jc\omega_{4}e^{j\theta_{4}}=0$$

where a, b and c represent the known length of the links. $\theta_{2}$ and $\omega_{2}$ are the angular position and speed of the input link, respectively.$\;\theta_{3}$, $\theta_{4}$, $\omega_{3}$ and $\omega_{4}$ are the angular positions and speeds of the coupler and output links, respectively. $e^{j\theta_{n}}$ is the Euler´s formula that is expressed by Eq. (2). For both Eqs. (1) and (2), j term is a complex number used to represent the vertical position of speed vectors in a complex plane.

(2) $$e^{j\theta_{n}}=\cos\theta +j\;\sin\theta$$

Solving Eq. (1), the expression for calculating the output angular velocity, $\omega_{4}$, is:

(3) $$\omega_{4}=\frac{a\omega_{2}}{c}\frac{\sin\left(\theta_{2}-\theta_{3}\right)}{\sin\left(\theta_{4}-\theta_{3}\right)}$$

Ball´s linear sliding speed, $v_{B}$, in the four-bar mechanism is then calculated by the following expression:

(4) $$v_{B}=r\omega_{4}$$

where r is the radius of the ball. An example of a ball´s sliding speed, $v_{B}$, function for an entire cycle of the electric motor (360°) is exemplified in Fig. 2c by using the geometrical features shown in Fig. 2b.

Limit positions of the rocker arm can be calculated by Eq. (5) and (6).

(5) $$\theta_{4\prime }=\cos^{-1}\left[\frac{c^{2}+d^{2}-{\left(a+b\right)}^{2}}{2cd}\right]$$

(6) $$\theta_{4\prime \prime }=\cos^{-1}\left[\frac{c^{2}+d^{2}-{\left(b-a\right)}^{2}}{2cd}\right]$$

Where θ_4′_ and θ_4″_ are the two limit positions of the rocker arm, *a* is the length of the crank, *b* is the length of the coupler, *c* is the length of the rocker and *d* is the length of the fixed bar.

The arc described for the rocker arm can be calculated by Eq. (7)

(7) $$\forall =\left|\theta_{4\prime }-\theta_{4\prime \prime }\right|$$

While the length stroke of the ball is calculated by Eq. (8).

(8) $$Arc\;Lenght=\frac{\forall \left(360^{\circ }\right)}{2\pi }\;R$$

where R represents the ball radius.

### Sample and slurry preparation

For the testing, the surface of the material samples should be polished to achieve the desired roughness prior testing. Typically, they are prepared to have Ra roughness between 0.02 and 0.1 ± 0.01 µm. On the other hand, the AISI 52100 steel balls must be subjected to special surface conditioning. They can be etched in a mixture of Nital solution (nitric acid (30 vol.%) and ethyl alcohol (70 vol.%)) during 30 s. It provides a modified surface with a pitted pattern with Ra roughness of 0.4 ± 0.05 μm, as required in the original test [6,13,14]. Other surface techniques for the pitting surface can be also employed. The material and ball samples must be cleaned meticulously with an appropriate solvent (e.g., alcohol or acetone) for eliminating most of contaminants that can alter the wear progression.

The slurry is suggested to be made of deionized water with a defined amount of suspended abrasive particles which is achieved by stirring. The most common abrasive particles used for micro-abrasion testing are angular F-1200 SiC particles with size in the range of 4–10 μm, so they are suggested for the testing. However, other kind of particles made of different materials, sizes and shapes can be used. The abrasive particles amount suggested for achieving the reproduction of micro-abrasion in the form of pure rolling abrasion is between 80 and 120 g per 100 ml of distilled water while less than 4 g per 100 ml are suggested for micro-abrasion in the form of grooving. The slurry must be agitated continuously during its application in the test and continuously dripped onto the ball, preferably in the closest region to the sliding contact, at a constant feed rate. The slurry runs off the ball surface by gravity effects and allows a continuous feeding of slurry to the sliding contact. Although controlling the number of particles in the slurry can be the main route to control the micro-abrasion type, it is suggested to conduct pretesting under different loads and ball frequency for guaranty the resulted wear patterns. Also, pretesting is needed to identify the sliding distance required to achieve a steady wear state of the material to be tested since short sliding distances can promote only wear under the running-in stage generating error in the wear rate results. For this, different trials at increasing sliding distance for the conditions required can be conducted to deploy the wear rate as function of sliding distance, and thus identify the steady wear state region, which is different for each material and test condition.

### Parameter choice

Some suggested parameters for micro-abrasion (rolling abrasion and grooving) testing under oscillating sliding are given in Table 1. Each condition tested should be replicated (≥ 3 times) using new samples and balls until reaching a wear diameter/volume standard deviation ≤15%, which is considered as acceptable for wear tests according to Ref. [14]. According to damage generated to the ball surface (sliding arc length) by wear during the test, it is suggested rotating the ball transversally at least every 1–20 m of sliding distance (depending on each material to be tested) using arc lengths between 10 and 20 mm.

**Table 1:** Suggested micro-abrasion test conditions for oscillating sliding.

Parameter		Micro-abrasion(rolling abrasion)	Micro-abrasion(Grooving)
Applied load [N]		0.1–1.5	0.1–1.5
Sliding distance [m]		4–60	4–60
Abrasive concentration in distilled water		80–120 g /100 ml	1–4 g / 100 ml
Slurry feed rate [g/min]		0.02–2	0.02–2
Frequency [Hz]		2.5–5	2.5–5
Oscillating sliding	Mean ball´s sliding speed [m/s]	0.05–0.1	0.05–0.1
	Max. ball´s sliding speed [m/s]	0.15	0.15
	Ball´s sliding arc length [mm]	10–20	10–20

### Wear quantification

**Fig. 3:**
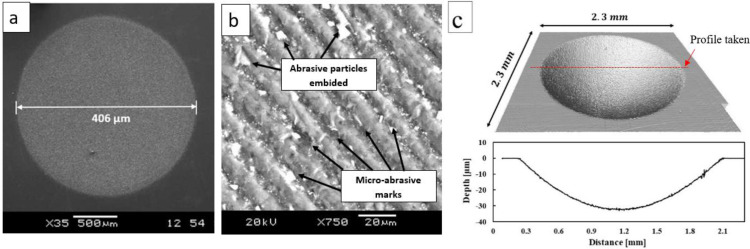
Examples of micro-abrasion wear scar analyses: (a) diameter measurement by SEM; (b) SEM image exhibiting detailed wear patterns; (c) wear scar scanned by optical profilometer (Brucker Contour GTK).

After micro-abrasion testing, the wear scars generated on the material samples are measured to determine the quantity of material removal by wear, and thus estimating wear coefficient/specific wear rate and wear resistance of the material. The scars can be measured either by optical microscopy, SEM or optical profilometry. Optical microscopy and SEM are useful to accurately measure the main parameters of the scar geometry, as shown in Fig. 3a, for example diameter,b, from a spherical cap scar, which may be used in Eq. (9) [6] to calculate the wear volume generated for a ball with radius, R. SEM is often used apart to analyze the wear features in much more detail, as shown in Fig. 3b. Otherwise, optical profilometry is advantageous because it can be used for scanning the entire wear scar, as illustrated in Fig. 3c, and estimate directly the wear volume through using a software. Having the wear volume,$\;V_{w}$, the specific wear rate, k, and the wear resistance, $\frac{1}{k}$, that is the inverse of the specific wear rate, for a certain sliding distance, S, and applied load, N, can be calculated by Eq. (10) [16].

(9) $$V_{w}=\frac{\pi b^{4}}{64R}\text{forb}<<R$$

(10) $$k=\frac{V_{w}}{SN}$$

### Procedure

To carry out a micro-abrasion test under oscillating sliding, the following steps are suggested:

(1) Prepare the material samples by polishing them till reaching the desired roughness.
(2) Make the surface modification of the balls to be used for the testing.
(3) Set up the micro-abrasion tester to generate the oscillation movement of the ball by adjusting the desired sliding arc length and frequency.
(4) Mount the sample and the ball to the tester.
(5) Clean meticulously both surfaces by using acetone preferably.
(6) Prepare the slurry with the selected abrasive particles concentration and disperse them in distilled water or another medium by stirring.
(7) Carry out to preliminary oscillatory tests to identify the steady wear rate state of the material under the conditions predefined and to determine the minimum sliding distance required for characterize the material under steady state.
(8) Conduct the micro-abrasion testing under suggested conditions.
(9) Perform the required repeats (≥ 3) till obtain a suggested wear volume standard deviation ≤15%.
(10) Apply the slurry at the defined feed rate and rotate transversely the ball during the test as suggested.
(11) Analyze the wear scar formed on the material sample.
(12) Estimate specific wear rate/coefficient, wear resistance, etc.

## Case study

A case study focused on the characterization of an ASTM F1537 CoCrMo alloy in terms of rolling abrasion and grooving under unidirectional and oscillating sliding, for comparison purposes, has been conducted for illustrating the present procedure (step-by-step) and presenting some results examples obtained by using this method. The test parameters for the case study are shown in Table 2.

**Table 2:** Test conditions for the case study.

Parameter		High abrasive particles concentration (HAPC)	Low abrasive particles concentration (LAPC)
Wear mode		Rolling abrasion	Grooving
Load [N]		1	1
Sliding distance [m]		25	25
Abrasive concentration		80 g /100 ml	4 g / 100 ml
Steel ball hardness [HV GPa]		3.7	3.7
Unidirectional sliding	Sliding speed [m/s]	0.1	0.1
Oscillating sliding	Mean sliding speed [m/s]	0.1	0.1
	Max. sliding speed [m/s]	0.15	0.15
	Arc length [mm]	12	12
	Frequency [Hz]	4.2	4.2

### Procedure details

**Fig. 4:**
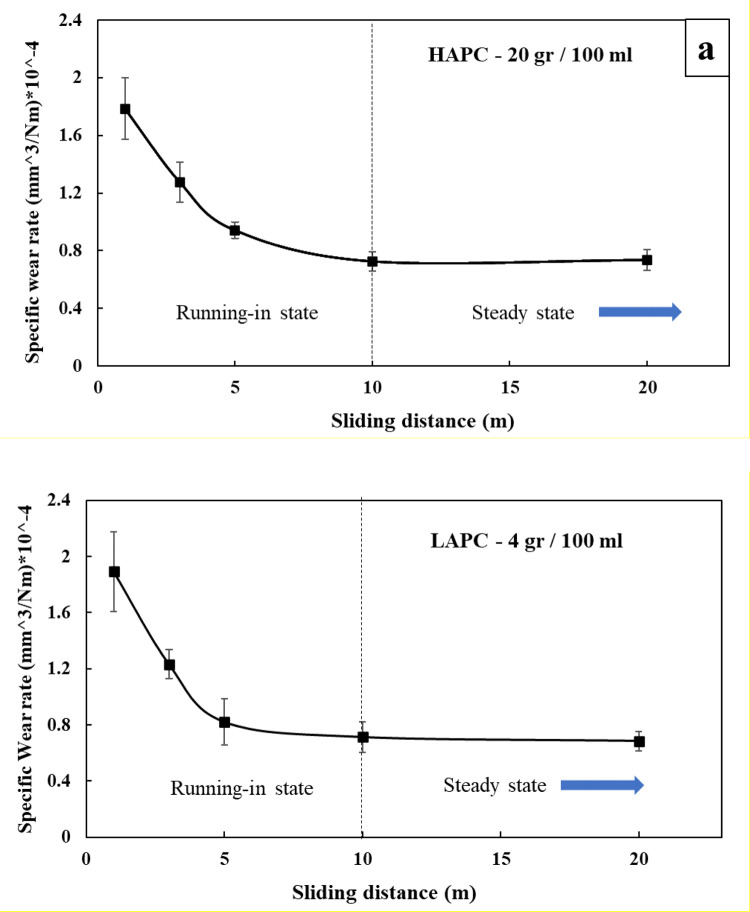
Steady wear state condition evaluation under oscillating sliding.
(8) A slurry feed rate of 0.5 g/min was applied by using a peristaltic pump fixed in the tester. The ball was rotated transversally each 20 and 2.75 m of sliding distance for the unidirectional and oscillating sliding tests, respectively.
(9) The experiment was replicated three times for each condition. A standard deviation of ≤15% was achieved for all tested conditions.
(10) Tested sample was dismounted and then cleaned with pressurized water to remove the remaining abrasive slurry. The surface of the sample was then cleaned again with acetone.
(11) The wear patterns generated were analyzed by optical microscopy. Fig. 5a–d show some representative wear scars obtained for each tested condition. As it was expected, multiple micro indentations with any evidence of a direction pattern and highly oriented grooving marks were the main characteristics identified in wear scars when HAPC and LAPC slurries were used in unidirectional micro-abrasion tests. These wear mechanisms are caused by rolling abrasion and grooving, respectively. Wear scars obtained using HAPC slurry in oscillating micro-abrasion tests are very similar to those obtained in unidirectional tests with same abrasive slurry condition. In contrast, when LAPC slurry is used in oscillating tests, a considerable number of micro indentations along with grooving marks can be observed. It is important to point out that micro indentations are a common characteristic in wear scars when the abrasive particles concentration is high. In this case, the presence of micro indentations could be attributed to the motion pattern at which particles are subjected in the oscillating motion of the counterpart, see a detailed discussion in Ref. [15].

(1) Circular flat samples (20 mm diameter and 5 mm thickness) made of ASTM F1537 CoCrMo alloy were prepared. The surfaces of all samples were polished to achieve a surface roughness (Ra) of 0.03 ± 0.01 µm.
(2) AISI 52100 steel balls of 25.4 mm of diameter were chosen as counterparts. The balls were subjected to chemical attack using Nital reagent (30% nitric acid - 70% alcohol) to achieve a pitted surface roughness (Ra) of 0.4 ± 0.05 µm.
(3) The tester was configured as illustrated in Fig. 2a, b using the same geometrical features for reproducing oscillating movement (12 mm arc length and 1.25 Hz frequency) of the ball.
(4) The samples were mounted on the tester using the corresponding holders.
(5) Both ball and flat specimen surfaces were cleaned with acetone.
(6) The abrasive slurry was made by dispersing F-1200 abrasive SiC particles (4–8 µm) in deionized water by magnetic stirring. Two different volume concentrations of abrasive particles in the slurries were used: 80 g/100 ml for rolling abrasion and 4 g/100 ml for grooving.
(7) Preliminary oscillatory tests were conducted using 1, 3, 5, 10 and 20 m of sliding distance for both high abrasive particle concentration (HAPC – 80 g/100 ml) and low abrasive particle concentration (LAPC - 4 g/100 ml) to identify the steady wear state of the material under the conditions predefined and to determine the minimum sliding distance required for characterizing the material under steady state. Each condition was replicated three times. The specific wear rate results of pretests are shown in Fig. 4. It was determined that at least 10 m of sliding distance are necessary to obtain a steady state wear behavior for this case. Finally, the tests were run under the conditions in Table 2.

**Fig. 5:**
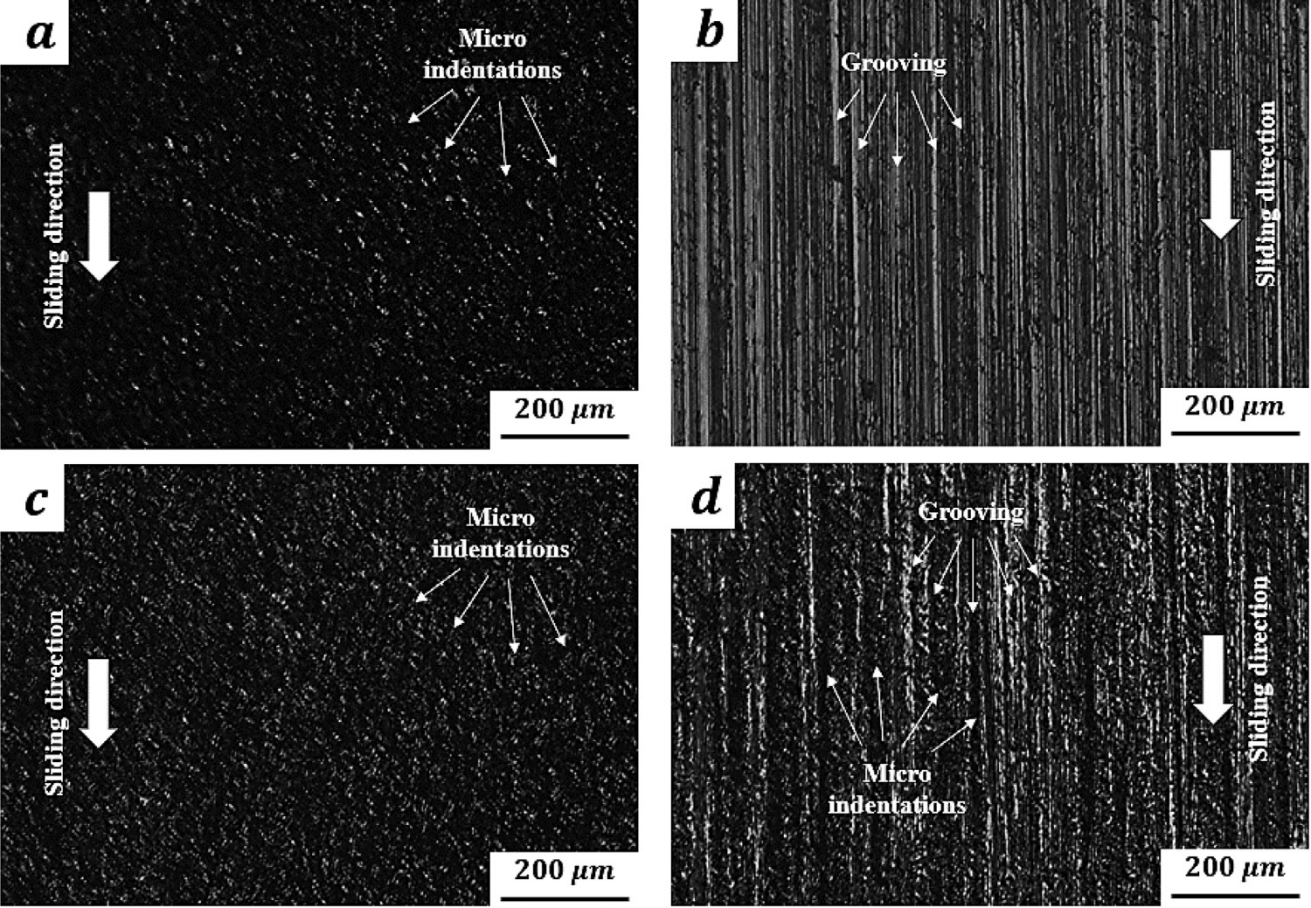
Representative wear patterns observed by optical microscopy: (a) rolling abrasion under unidirectional sliding; (b) grooving under unidirectional sliding; (c) rolling abrasion under oscillating sliding; (d) grooving under oscillating sliding.
(12) An example of the wear craters geometry generated by both rolling abrasion and grooving tests is illustrated in Fig. 3a and c. The wear craters mean diameter, b, of the wear scars was measured by optical microscopy to calculate the volume loss and specific wear rate according to Eqs. (9) and (10). The specific wear rate results are shown in Fig. 6. Wear rate differences can be seen by comparing the results for HAPC and LAPC and for unidirectional and oscillating sliding motions.

**Fig. 6:**
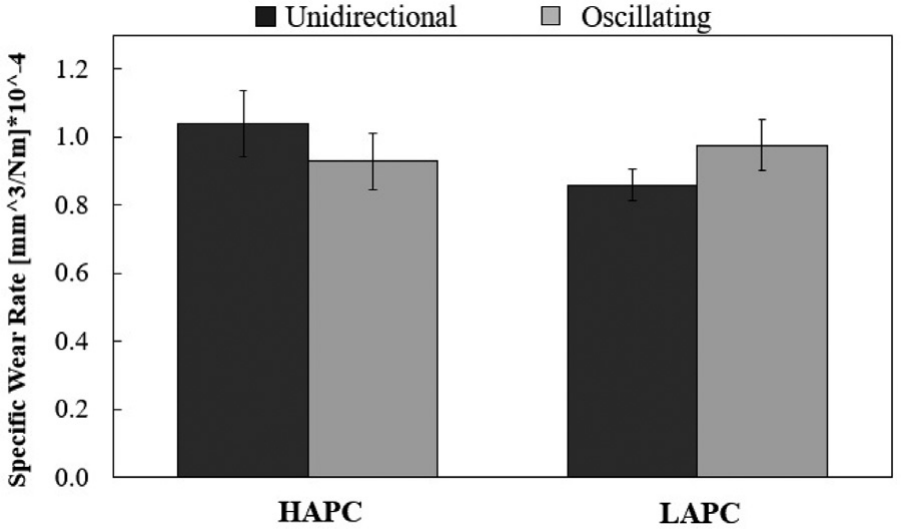
Comparison of specific wear rate results obtained by unidirectional and oscillating micro abrasion tests using HAPC and LAPC slurries.

## Declaration of Competing Interest

The authors declare no conflict of interest.
